# Early lysosomal maturation deficits in microglia triggers enhanced lysosomal activity in other brain cells of progranulin knockout mice

**DOI:** 10.1186/s13024-018-0281-5

**Published:** 2018-09-04

**Authors:** Julia K. Götzl, Alessio-Vittorio Colombo, Katrin Fellerer, Anika Reifschneider, Georg Werner, Sabina Tahirovic, Christian Haass, Anja Capell

**Affiliations:** 1 Chair of Metabolic Biochemistry, Biomedical Center (BMC), Faculty of Medicine, Ludwig-Maximilians-Universität München, 81377 Munich, Germany; 2 German Center for Neurodegenerative Diseases (DZNE) Munich, 81377 Munich, Germany; 3 Munich Cluster for Systems Neurology (SyNergy), 81377 Munich, Germany

**Keywords:** Frontotemporal lobar degeneration, Microglia, Neurodegeneration, Progranulin, Lysosome, Cathepsin

## Abstract

**Background:**

Heterozygous loss-of-function mutations in the progranulin gene (*GRN*) lead to frontotemporal lobar degeneration (FTLD) while the complete loss of progranulin (PGRN) function results in neuronal ceroid lipofuscinosis (NCL), a lysosomal storage disease. Thus the growth factor-like protein PGRN may play an important role in lysosomal degradation. In line with a potential lysosomal function, PGRN is partially localized and processed in lysosomes. In the central nervous system (CNS), PGRN is like other lysosomal proteins highly expressed in microglia, further supporting an important role in protein degradation. We have previously reported that cathepsin (Cat) D is elevated in *GRN*-associated FTLD patients and *Grn* knockout mice. However, the primary mechanism that causes impaired protein degradation and elevated CatD levels upon PGRN deficiency in NCL and FTLD remains unclear.

**Methods:**

mRNA expression analysis of selected lysosomal hydrolases, lysosomal membrane proteins and autophagy-related genes was performed by NanoString nCounter panel. Protein expression, maturation and in vitro activity of Cat D, B and L in mouse embryonic fibroblasts (MEF) and brains of *Grn* knockout mice were investigated. To selectively characterize microglial and non-microglial brain cells, an acutely isolated microglia fraction using MACS microbeads (Miltenyi Biotec) conjugated with CD11b antibody and a microglia-depleted fraction were analyzed for protein expression and maturation of selected cathepsins.

**Results:**

We demonstrate that loss of PGRN results in enhanced expression, maturation and in vitro activity of Cat D, B and L in mouse embryonic fibroblasts and brain extracts of aged *Grn* knockout mice. Consistent with an overall enhanced expression and activity of lysosomal proteases in brain of *Grn* knockout mice, we observed an age-dependent transcriptional upregulation of certain lysosomal proteases. Thus, lysosomal dysfunction is not reflected by transcriptional downregulation of lysosomal proteases but rather by the upregulation of certain lysosomal proteases in an age-dependent manner. Surprisingly, cell specific analyses identified early lysosomal deficits in microglia before enhanced cathepsin levels could be detected in other brain cells, suggesting different functional consequences on lysosomal homeostasis in microglia and other brain cells upon lack of PGRN.

**Conclusions:**

The present study uncovers early and selective lysosomal dysfunctions in *Grn* knockout microglia/macrophages. Dysregulated lysosomal homeostasis in microglia might trigger compensatory lysosomal changes in other brain cells.

**Electronic supplementary material:**

The online version of this article (10.1186/s13024-018-0281-5) contains supplementary material, which is available to authorized users.

## Background

Pathogenic mutations in the progranulin gene (*GRN*) are genetically linked to frontotemporal lobar degeneration (FTLD) and a rare adult-onset form of neuronal ceroid lipofuscinosis (NCL) [1–4]. Haploinsufficiency caused by non-sense and a few missense mutations result in *GRN-*associated FTLD (FTLD/*GRN*) [1, 2, 5–7] while homozygous loss-of-function *GRN* mutation carriers, who completely loose progranulin (PGRN) expression, develop NCL [3, 4]. Gene mutations causative for the different NCL forms are mostly associated with the lysosomal degradation pathway [8], indicating that PGRN may also be involved in lysosomal function. Indeed, *Grn* knockout mice are characterized by an increase of lysosomal proteins, NCL-like storage material, lipofuscinosis and an accumulation of the autophagy markers ubiquitin and p62 [3, 9–14]. Beside the genetic link between PGRN and lysosomal disorders [3, 4], there is also increasing evidence supporting lysosomal dysfunction in FTLD [15]. Hallmarks of all FTLD/*GRN* patients are cytoplasmic inclusions of hyperphosphorylated TAR DNA binding protein (TDP)-43 co-localizing with ubiquitin and p62 [16, 17]. Furthermore, FTLD/*GRN* patients show symptoms typically associated with NCL, like retinal thinning, lipofuscin and NCL-like storage material deposits [18–21]. Finally, challenging lysosomes by starvation, sucrose treatment, inhibition of vacuolar ATPase or alkalizing drugs causes an increase in PGRN production [21–23]. PGRN is a multifunctional complex glycosylated protein, which can be secreted as a growth factor into the extracellular space [24–26]. Secreted PGRN is also processed to granulin peptides by several different proteases including matrix metalloproteinases − 9 and − 14 [27, 28], a disintegrin and metalloproteinase with thrombospondin motif 7 (ADAMTS-7) [29], neutrophil elastase, proteinase 3 [25, 30], and intracellular by cathepsin L (Cat L) [31, 32]. Additionally, PGRN can be transported to lysosomes using two distinct transport pathways; one mediated by the sortilin receptor [33] and the other one via complex formation with prosaposin. Prosaposin is then transported to lysosomes by binding to the mannose-6-phosphate receptor or the low density lipoprotein receptor-related protein 1 [34]. Recent evidence indicates that lysosomal localized PGRN [33, 35–37] may function in lysosomal homeostasis and autophagy [38–40]. Additionally, granulin peptides, which are generated within lysosomes [31, 32, 41], might affect the lysosomal function [38].

To further understand if and how the loss of PGRN may cause lysosomal dysfunction we searched for changes in protein expression, maturation and enzymatic activity of a subset of lysosomal cathepsins in microglia and other brain cells. Microglia, which are the main source of PGRN in the brain, showed a severe impairment of lysosomal cathepsin expression and maturation in the absence of PGRN whereas the remaining brain cells showed increased cathepsin processing and maturation. We therefore propose different effects on lysosomal function upon loss of PGRN in microglia and other brain cells like astrocytes and neurons.

## Methods

### Animal experiments and mouse brain tissue

All animal experiments were performed in accordance with local animal handling laws. Mice were sacrificed by CO_2_ inhalation. Brain tissue was obtained from the *Grn*^**−/−**^ mouse strain generated by Kayasuga and colleagues [42].

### Isolation of adult primary microglia, neurons and astrocytes

Primary microglia were isolated from adult mouse brain using MACS Technology (Miltenyi Biotec) according to manufacturer’s instructions. Briefly, brain cortices were dissected, and after removal of meninges dissociated by enzymatic digestion using the Neural Tissue Dissociation Kit P (Miltenyi Biotec). CD11b-positive microglia were magnetically labelled with CD11b MicroBeads, loaded onto a MACS Column (Miltenyi Biotec) and subjected to magnetic separation. Isolated microglia and the microglia depleted fraction were snap frozen in liquid nitrogen and stored at − 80 °C until further biochemical analysis. To determine PGRN expression in different brain cell types, microglial, neuronal and astrocytic cells, the dissected mouse brain, after removal of meninges, was dissociated by using the Adult Brain Dissociation Kit (Miltenyi Biotec). A single cell suspension was generated according to the manufactures instructions with the exception that the mouse brain was mechanically dissociated by using in sequence, three fire-polished Pasteur pipettes with decreasing tip diameters. The single cell suspension of five mice were combined and separated in three aliquots for isolating microglia cells (Anti-CD11b MicroBeads, Miltenyi Biotec), astrocytes (Anti-ACSA-2 MicroBeads, Miltenyi Biotec) and neurons (Neuron Isolation Kit, Miltenyi Biotec) according to the manufactures instructions. Isolated cells were snap frozen in liquid nitrogen and stored at − 80 °C until further biochemical analysis.

### Generation and culturing of mouse embryonic fibroblasts (MEF) lines

*Ctsd*^**−/−**^ MEF were provided by Dr. Paul Saftig and generated from the *Ctsd* knockout mouse strain [43]. For *Grn*^**−/−**^ MEF, timed pregnant female of a heterozygous *Grn* mating was sacrificed by CO_2_ inhalation, the embryos (E15) were processed and MEF were separated by digestion with trypsin-EDTA [44]. MEF cells with the same genotype derived from littermates were pooled and immortalized by serial (3 T3) passaging [44]. From immortalized MEF either single cell clones were isolated or pools were generated. For rescue of PGRN deficiency, *Grn*^**−/−**^ MEF were transfected with m*Grn* cloned into the HindIII and XhoI site of pcDNA3.1 (Hygro+) and selected with hygromycin B at 50 mg/mL (Invitrogen). Single cell clones were analyzed for PGRN expression. MEF were cultured in DMEM with Glutamax I (Invitrogen) supplemented with 10% fetal calf serum (Invitrogen) and penicillin/streptomycin (PAA Laboratories).

### Quantitative NanoString nCounter gene expression assay

NanoString nCounter technology allows expression analysis of multiple genes from a single sample. We generated an nCounter panel for analyzing gene expression of 45 lysosomal and autophagy- related genes [45–48] and 5 housekeeping genes. Total RNA was extracted from aliquots of powdered mouse brain samples using QIAshredder and RNeasy Mini Kit (Qiagen). 100 ng RNA per brain was used for gene expression analysis. The NanoString panel measurement and evaluation was done at Proteros Biostructures GmbH, Martinsried, Germany. Gene expression levels in each sample were normalized against the geometric mean of four housekeeping genes including *Cltc, Hprt, Pgk1* and *Tubb5* using *nSolver*™ *Analysis Software, version 3.0* (NanoString Technologies, Inc.) *Gusb* was excluded because of significant changes of expression in *Grn*^**−/−**^ mice. Based on the normalized gene expression levels of *Grn*^**−/−**^ and *Grn*^+/+^ (*n* = 3), statistical significance was determined by the unpaired, two-tailed student’s t-test.

### Quantitative real time PCR (qRT-PCR)

Approximately 10–20 mg of powdered mouse brain homogenates were subjected to total RNA preparation using the QIAshredder and RNeasy Mini Kit (Qiagen) according to manufacturer’s instructions. 2 μg of RNA was reverse transcribed into cDNA using M-MLV reverse transcriptase (Promega) and oligo(dT) primers (Life Technologies). The following primer sets from Integrated DNA Technologies were used: mouse *Ctsd* Mm.PT.53a.17202883 (Exon boundary 3 to 4), mouse *Ctsb* Mm.PT.53a.7639164 (Exon boundary 4 to 5), mouse *Ctsl* Mm.PT.58.9857472 (Exon boundary 7 to 8), mouse *App* Mm00431827_m1 (Applied Biosystems) and mouse *Gapdh* Mm.PT.39a.1 (Exon boundary 2 to 3). cDNA levels were quantitatively determined in triplicates using TaqMan assays on a 7500 Fast Real-Time-PCR System (Applied Biosystems). All cDNA levels were normalized to *Gapdh* cDNA and relative transcription levels of the respective sequences were analyzed using the comparative delta Ct method (7500 Software V2.0.5, Applied Biosystems, Life Technologies).

### Antibodies

The following primary antibodies were used for immunoblotting: mouse monoclonal anti-β-actin antibody (Sigma-Aldrich; 1:10,000), mouse monoclonal anti-α-tubulin antibody (Sigma-Aldrich; 1:5,000), goat anti-cathepsin D (sc-20) antibody (Santa Cruz Biotechnology; 1:500), goat anti-cathepsin B (AF965) antibody (R&D Systems; 0,1 μg/ml), goat anti-cathepsin L (AF1515) antibody (R&D Systems; 1 μg/ml), goat anti-cathepsin S (M-19) antibody (Santa Cruz Biotechnology; 1:200), rat anti-PGRN (8H10) antibody (1:50) [18]; rabbit anti-GFAP antibody (Dako; 1:5,000), rabbit anti-Iba1 antibody (Dako; 1:1000), rabbit anti-neuronal class III ß-Tubulin (Tuj1) antibody (BioLegend; 1:10,000), rabbit anti-p62/SQSTM1 antibody (MBL; 1:1,000), mouse anti-ubiquitin (P4D1) antibody (Santa Cruz Biotechnology; 1:1000), rabbit anti-APP (Y188) antibody (Abcam, 1:2,000) rat monoclonal anti-mLamp1 antibody clone 1D4B (developed by J. Thomas August, distributed by Developmental Studies Hybridoma Bank, NICHD, maintained by the University of Iowa, Department of Biology; 1:200), goat polyclonal anti-saposin D antibody (1:1,000) [49], and rabbit anti-LC3BB/MAP1LC3B antibody (Novus Biologicals; 2 μg/ml). The following secondary antibodies were used: horseradish peroxidase-conjugated donkey anti-goat IgG (Santa Cruz Biotechnology; 1:5,000), anti-mouse IgG (Promega; 1:10,000), anti-rabbit IgG (Promega; 1:20,000), goat anti-rat IgG + IgM (L + M) (Dianova; 1:5,000) and generated mouse anti-rat IgG2c (1:1,000).

### Protein analysis and immunoblotting

For microglia, neurons and astrocytes cell pellets were lysed in RIPA buffer [18] supplemented with protease inhibitor cocktail (Sigma-Aldrich) and phosphatase inhibitor (Roche Applied Science) and centrifuged for 30 min, 15,000 x g, 4 °C. MEF cells were lysed in RIPA buffer (150 mM NaCl, 50 mM TRIS pH 8.0; 0.1% SDS, 1% NP40, 0.5% Sodiumdeoxycholat) supplemented with Benzonase (Novagen), protease inhibitor cocktail (Sigma-Aldrich) and phosphatase inhibitor (Roche Applied Science) and centrifuged for 30 min, 15,000 x g, 4 °C. The protein concentration of the supernatant was determined using the BCA protein assay (Pierce, Thermo Scientific) and equal amount of protein were separated by SDS-PAGE and transferred onto polyvinylidene difluoride membranes (Immobilon-P, Merck Millipore). For the detection of C-terminal fragments (CTF) of the amyloid precursor protein (APP) and saposin D the proteins were transferred onto nitrocellulose membranes (Protran BA85, GE Healthcare Lifesciences) and heated in PBS. Proteins of interest were detected by the indicated primary antibodies followed by horseradish peroxidase-conjugated secondary antibodies and ECL (Amersham Western Blotting Detection reagent, GE Healthcare Lifesciences) or ECL Plus (Pierce ECL Plus Western Blotting Substrates, Thermo Scientific). For the quantitatively analysis, images were taken by a Luminescent Image Analyzer LAS-4000 (Fujifilm Life Science, Tokyo, Japan) and evaluated with the Multi GaugeV3.0 software (Fujifilm Life Science, Tokyo, Japan).

### Cathepsin activity assay

MEF cell pellets or aliquots of powdered mouse brain tissues were used for cathepsin D, B and L fluorescence based activity assays (Abnova). The samples were homogenized in the appropriate lysis buffer provided by the manufacturer and incubated for 10 min (MEF cell lysates) or 20 min (brain lysates) on ice, followed by a 5 min (MEF cell lysates) or 20 min (brain lysates) centrifugation at 15,000 x g, 4 °C. The protein concentration was determined by BCA protein assay (Pierce, Thermo Scientific) and equal amounts of protein were used for the activity assays. The assays were performed in black 96-well plates (FluoroNunc) at 37 °C for 20 min according to the manufacturer’s protocol. Cleavage of the quenched fluorescence substrate was continuously measured as increase of fluorescence signal by Fluoroskan Ascent FL plate reader (Labsystems). The relative enzyme activity was calculated for a period of time with linear substrate turnover.

### Metabolic labeling and protein turn over

To analyze protein turnover, MEF at 70–80% of confluency were starved for 1 h in methionine-, cysteine- and serum-free minimal essential medium (Invitrogen) and subsequently metabolically pulse-labeled with 18.5 MBq ^35^S-methionine/cysteine (Met-S35-label, Hartmann Analytic) in methionine-, cysteine- and serum free medium for 1 h, followed by indicated chase periods in the presence of a 5-fold excess of unlabeled methionine. Cell lysates were prepared and labeled proteins were precipitated with 5% TCA for 1 h at 4 °C, followed by 30 min centrifugation at 13,000 rpm, 4 °C. Pellets were washed twice with 80% acetone, dried at RT and resuspended in 50 μl sample buffer. Remaining radioactive-labeled proteins were measure in triplicates in liquid scintillation counter (Tri-Carb 2810, Perkin Elmer).

### Statistical analysis

For statistical analysis the unpaired, two-tailed student’s t-test was performed when two groups of samples (wt and ko) were compared, for comparison of more than two groups, one-way ANOVA with Dunnett’s post hoc test was used and statistical significance was set at *, *p* < 0.05; **, *p* < 0.01; ***, *p* < 0.001; and ****, *p* < 0.0001.

## Results

### Subtle changes in lysosomal and autophagy-related gene expression in total brain of *Grn*^−/−^ mice

**Fig. 1:**
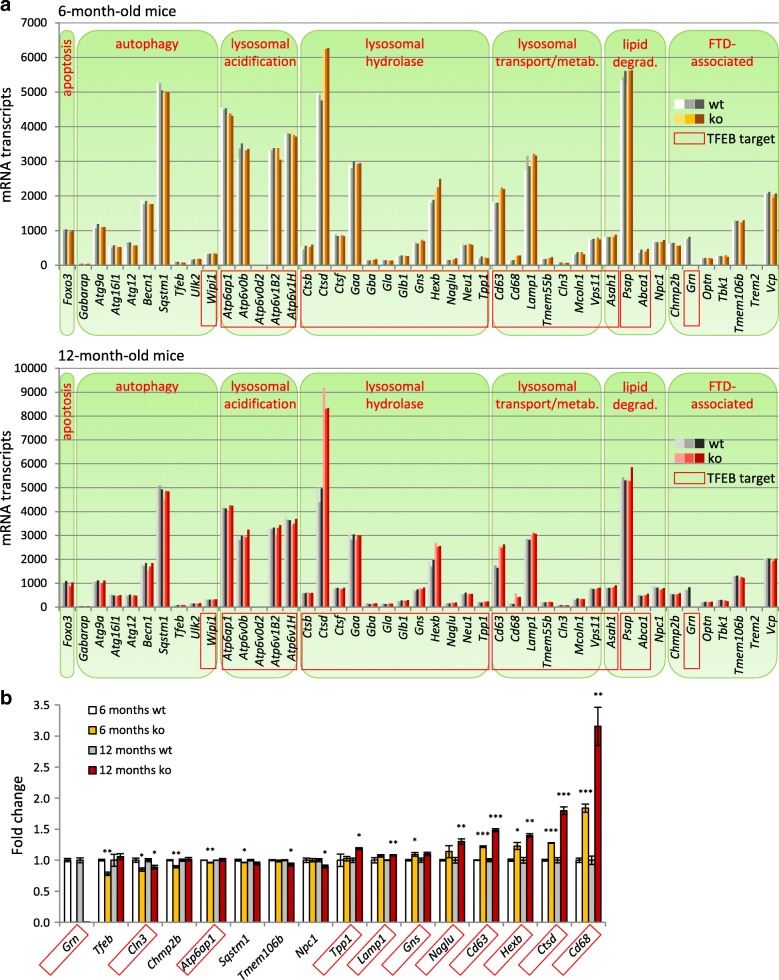
Minor alterations in expression of lysosomal and autophagy-related genes in brain of *Grn*^−/−^ mice. **a** mRNA expression for 45 selected genes associated with the lysosome-autophagy degradation pathway [45, 46] in brain of *Grn*^+/+^ (wt) and *Grn*^**−/−**^ (ko) mice at 6 and 12 months of age (for original data see Additional file 2). Genes are grouped by their function within these pathways or by their FTLD-association. Previously identified TFEB targets are labeled by red boxes [46, 47]. *N* = 3 mice per group, notice the low expression differences between individual mice and between 6- and 12-month-old mice. *Trem2* and *Atp6v0d2* were below the detection limit. **b** Fold change of gene expression which show at least one significand change either at 6 or 12 months of age. Data were normalized to the corresponding mean value of *Grn*^*+/+*^ (wt) mice and are shown as mean ± SD. For statistical analysis the unpaired, two-tailed student’s t-test was used (*n* = 3) (*, *p* < 0.05; **, *p* < 0.01; ***, *p* < 0.001)

Accumulating evidence indicates that PGRN plays a critical role for lysosomal integrity and function. To obtain insights into the role of PGRN in autophagic/lysosomal protein degradation pathways we performed on whole brain extract a NanoString based mRNA expression analysis of selected lysosomal and autophagy-related genes [45] (Fig. 1a). Surprisingly, expression of only very few genes was significantly altered in the brain of 6- and 12-month-old *Grn*^**−/−**^ mice (Fig. 1a, b). Only eight genes, among which are three genes encoding the lysosomal membrane proteins *Cd68*, *Cd63* and *Lamp1*, show a significantly elevated expression in *Grn*^**−/−**^ mice. Additionally, the lysosomal hydrolases *hexosaminidase subunit β* (*Hexb*) and *cathepsin D* (*Ctsd*) showed the strongest and age-dependent increase in *Grn*^**−/−**^ mice (Fig. 1b). However, no general elevation of lysosomal and autophagy-related gene expression regulated by transcription factor EB (TFEB) [46, 50] was observed in *Grn*^**−/−**^ mice brain. Thus, although *Grn*^**−/−**^ mice recapitulate important pathological features of NCL, expression of lysosomal genes is not overtly affected.

### Elevated cathepsin maturation and activity in PGRN deficient mouse brain

**Fig. 2:**
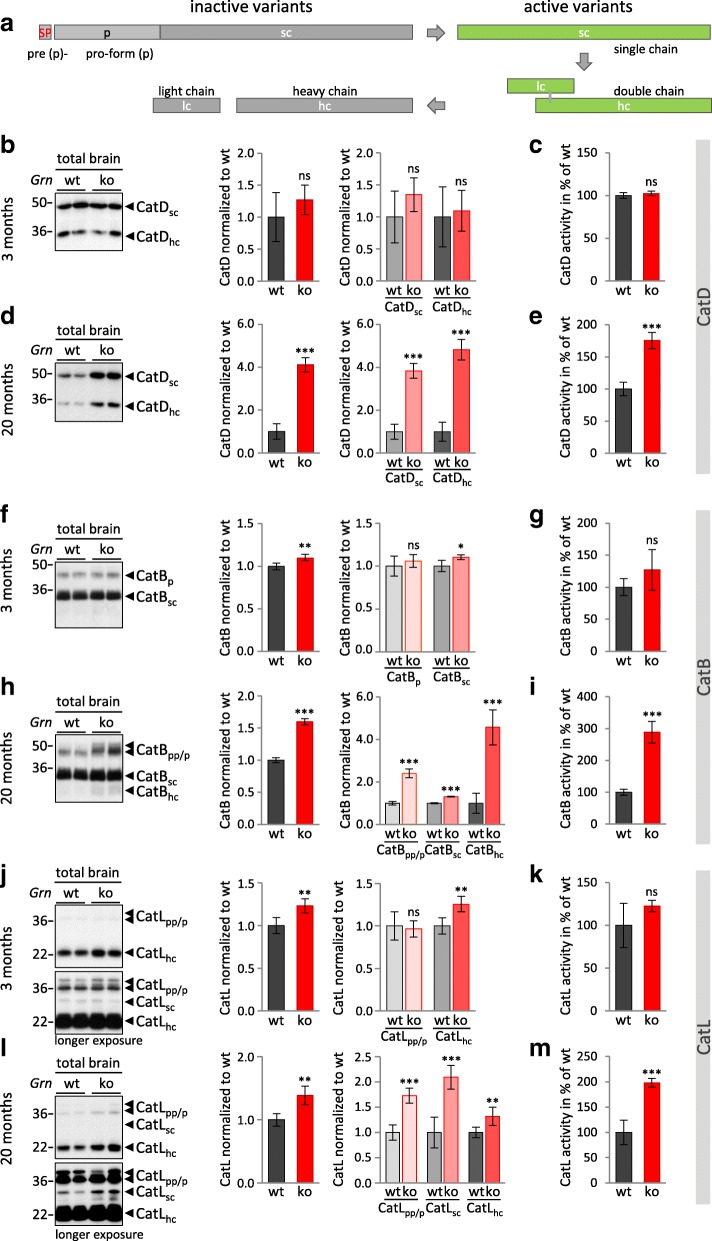
Altered protein expression, maturation and activity of cathepsins in brain of *Grn*^−/−^ mice. **a** Schematic presentation of cathepsin maturation [53]. Cathepsins (Cat) are synthesized as an inactive pre-pro-form (pp), translocated into the endoplasmic reticulum (ER) by signal peptide (SP). After SP removal the pro-form (p) becomes co-transitionally modified and is transported to lysosomes predominantly via the manose-6-phosphate pathway. With increasing acidification, the pro-peptide is removed, either autocatalytically or by other enzymes, leading to an active single chain variant (sc, green). For most cathepsins this sc-variant can be further proteolytically processed to a heavy (hc) and a light chain (lc), as long as hc and lc are linked by disulfide bridges or hydrophobic interaction the double chain variant remains active (green) but will be inactivated by separation of hc and lc. Representative blots of brain lysate from 3-month-old and 20-month-old *Grn*^+/+^ (wt) and *Grn*^**−/−**^ (ko) mice probed for cathepsin D (CatD) (**b, d**) cathepsin B (CatB) (**f, h**) and cathepsin L (CatL) (**j, l**). The molecular weight standards in kilo Daltons (kDa) are indicated on the left side of the blots. Quantification of blots for total cathepsin or maturation variants normalized to *Grn*^+/+^ are shown as mean ± SD. *N* = 5 mice per genotype (**b, d, f, h, j, l)**. Statistical significance was set at *, *p* < 0.05; **, *p* < 0.01; and ***, *p* < 0.001; ns, not significant using an unpaired, two-tailed student’s t-test**.** In vitro enzyme activity of CatD, CatB and CatL in lysates of mouse brains used for immunoblot analysis. Equal amounts of enzyme optimized brain lysates from *Grn*^+/+^ and *Grn*^**−/−**^ (*n* = 3–5) mice were incubated with quenched fluorogenic substrate (**c, e, g, i, k, m**). The increase of fluorescence signal was continuously measured and for a linear turnover time period normalized to *Grn*^+/+^ set as 100% activity, mean ± SD. Statistical significance was set at ***, *p* < 0.001 and ns, not significant using unpaired, two-tailed student’s t-test

We have previously shown that cathepsin D (CatD) is elevated in brain of young *Grn*^**−/−**^ mice and that CatD accumulation further increases with age [18]. These findings have been confirmed [12, 51], but the origin of CatD increase remains mainly unclear and cannot simply be explained as a compensation phenomenon in response to general reduced expression of lysosomal enzymes (Fig. 1). To address whether elevated *Ctsd* mRNA levels (Fig. 1b; Additional file 1: Figure S1) translate into increased protein levels and result in enhanced enzyme activity, we analyzed protein expression, maturation and in vitro activity of CatD in brain of 3- and 20-month-old mice (Fig. 2b-e). To further monitor lysosomal activity in *Grn*^**−/−**^ mice we also investigated protein expression, maturation and catalytic activity of two additional cathepsins, namely CatB (Fig. 2f-i) and CatL (Fig. 2j-m). Both lysosomal cysteine proteases have been associated with PGRN metabolism. CatL might be directly involved in lysosomal processing of PGRN into granulins [31] and CatL and CatB cleave and inactivate the secretory leucoprotease inhibitor (SLPI) which protects extracellular PGRN from processing [52]. Maturation and activation of most cathepsins follows a unified processing pathway generating active single and double chain variants (Fig. 2a) (reviewed in [53]). In 3-month-old *Grn*^**−/−**^ mice, CatD expression, maturation and activity was unchanged (Fig. 2b, c; Additional file 1: Figure S1). In aged mice (20 months) active single chain CatD (CatD_sc_) as well as further processed heavy chain CatD (CatD_hc_) are about 4- to 5-fold increased (Fig. 2d). This is accompanied by a 1.75-fold elevated proteolytic activity in *Grn*^**−/−**^ mice brain (Fig. 2e). In contrast to the robust increase of the CatD protein levels, we could only detect a 2-fold increase of mRNA (Additional file 1: Figure S1) suggesting posttranscriptional regulatory mechanisms. CatB and CatL are slightly elevated in young *Grn*^**−/−**^ mice (Fig. 2f, j) but no significant change in their catalytic activity was observed (Fig. 2g, k). Their mRNA levels were not altered (CatB) or only slightly elevated (CatL) (Additional file 1: Figure S1). In aged *Grn*^**−/−**^ mice, CatB and CatL expression, processing and activity were elevated (Fig. 2h, i, l, m; Additional file 1: Figure S1). Thus, against the expectations that lysosomal activity might be decreased in *Grn*^**−/−**^ mice, their proteolytic in vitro activity is elevated in total brain lysates.

### Increased activity of cathepsins in mouse embryonic fibroblasts (MEF) lacking PGRN

**Fig. 3:**
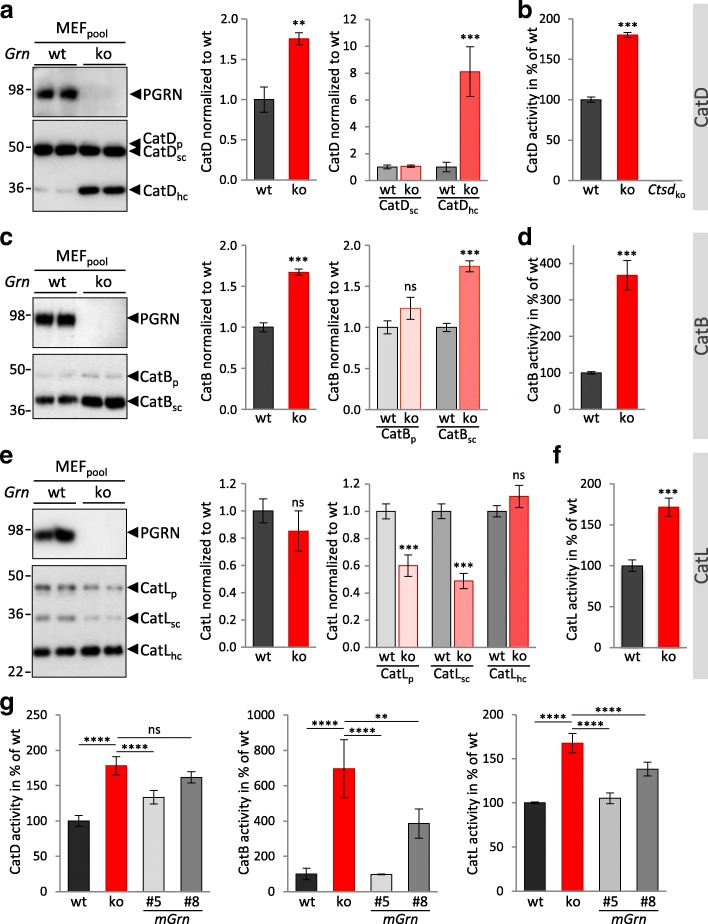
PGRN loss alters maturation and elevates activity of cathepsins in MEF. **a** CatD expression and maturation in MEF_pool_ *Grn*^+/+^ (wt) and *Grn*^**−/−**^ (ko) shown in representative immunoblots. The pro-form CatD_p_, single chain form CatD_sc_, and heavy chain form CatD_hc_ are indicated. Bar graphs show the quantification of blots for total CatD or maturation variants normalized to *Grn*^+/+^. **b** CatD activity measured as cleavage of a quenched fluorogenic substrate. The increase of fluorescence signal was continuously measured and during a linear turnover time period normalized to *Grn*^+/+^. Note that extracts of CatD deficient MEF (*Ctsd*_ko_) show no CatD activity and therefore confirm the specificity of the assay. **c** CatB expression and maturation in MEF_pool_ *Grn*^+/+^ (wt) and *Grn*^**−/−**^ (ko) shown in representative immunoblots. The pro-form CatB_p_, single chain form CatB_sc_ are indicated. Bar graphs show the quantification of blots for total CatB or maturation variants normalized to Grn^+/+^. **d** CatB activity normalized to *Grn*^+/+^. **e** CatL expression and maturation in MEF_pool_ *Grn*^+/+^ (wt) and *Grn*^**−/−**^ (ko) shown in representative immunoblots. The pro-form CatL_p_, single chain form CatL_sc_, heavy chain form CatL_hc_ are indicated. Bar graphs show the quantification of blots for total CatL or maturation variants normalized to *Grn*^+/+^. **f** CatL activity normalized to *Grn*^+/+^. **g** PGRN deficient MEF were stably transfected with mouse PGRN (mGrn) and low PGRN expressing single cell clones (#5, #8) were analyzed for CatD, CatB and CatL in vitro activity. Notice that very low expression of PGRN (#8) lowers cathepsin activities and thereby partially rescues the phenotype of the *Grn*^**−/−**^ MEF, while the higher expressing clone (#5) allows a full rescue for CatB and CatL. The molecular weight standards in kilo Daltons (kDa) are indicated on the left side of the blots. All bar graphs are shown as mean ± SD. Statistical significance was set at *, *p* < 0.05; **, *p* < 0.01; ***, *p* < 0.001; and ****, *p* < 0.0001 with ns as not significant using **a-f** unpaired, two-tailed student’s t-test (*n* = 3), **g** one-way ANOVA with Dunnett’s post hoc test (*n* = 3–6)

**Fig. 4:**
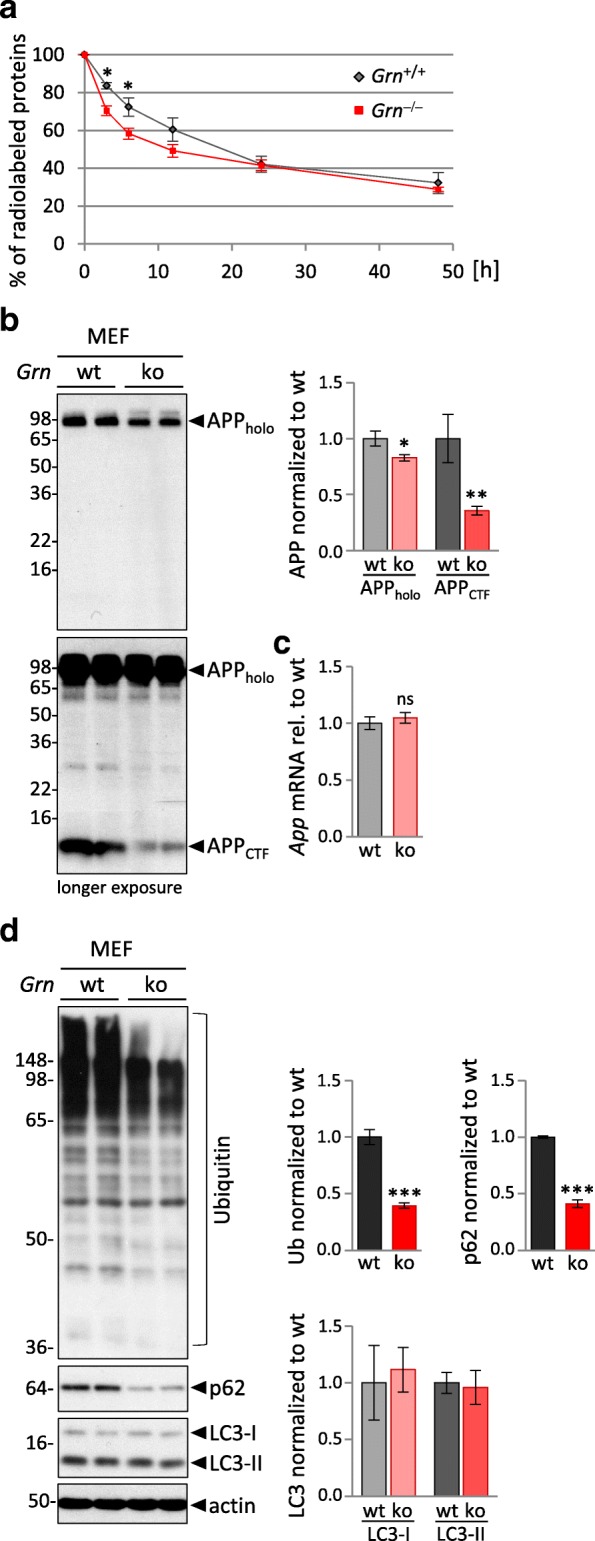
Elevated lysosomal activity results in enhanced fast protein degradation in *Grn*^−/−^MEF. **a** Turnover of ^35^S-methionine radiolabeled proteins. MEF at 70–80% confluency were metabolically pulse-labeled with ^35^S-methionine/cysteine for 1 h, followed by indicated chase periods. Radioactivity of ^35^S- labeled proteins at chase time point 0 h was set to 100% and remaining radioactive-labeled proteins at later chase points were normalized to the initial radioactivity at time point 0 h. For statistical analysis the unpaired, two-tailed student’s t-test was used to compare *Grn*^**−/−**^ to *Grn*^+/+^ MEF (*n* = 5), (*, *p* < 0.05). **b** APP holoprotein (APP_holo_) and C-terminal fragments (APP_CTF_) detected by immunoblotting of MEF_pool_ *Grn*^+/+^ (wt) and *Grn*^**−/−**^ (ko) lysates. The molecular weight standards in kilo Daltons (kDa) are indicated on the left side of the blots. Bar graphs show the quantification of the blots for APP_holo_ and APP_CTF_ normalized to *Grn* ^+/+^ (wt) as mean ± SD. **c** Quantification of *App* mRNA of MEF_pool_ *Grn*^**−/−**^ (ko) normalized to *Grn*^+/+^ (wt) as mean ± SD. **d** Ubiquitin (Ub), p62, LC3-I and LC3-II detected by immunoblotting of MEF_pool_ *Grn*^+/+^ (wt) and *Grn*^**−/−**^ (ko) lysates. Bar graphs show the quantification of the blots normalized to wt as mean ± SD. **b-d** For statistical analysis the unpaired, two-tailed student’s t-test was used to compare ko to wt cells (n = 3) (*, *p* < 0.05; **, *p* < 0.01; ***, *p* < 0.001; ns, not significant)

MEF generated from *Grn*^+/+^ show a robust localization of PGRN in lysosomes (Additional file 1: Figure S2a), while MEF generated from *Grn*^**−/−**^ littermates show an increase of LAMP1 and an accumulation of saposin D, which is in line with our previous observations in brains of *Grn*^**−/−**^ mice and FTLD/GRN patients [18] (Additional file 1: Figure S2b). We next examined the in vitro activity and maturation of CatD, CatB and CatL in *Grn*^**−/−**^ and *Grn*^+/+^ MEF (Fig. 3). In *Grn*^**−/−**^ MEF the overall CatD level was 1.7-fold elevated. Moreover, CatD_hc_ was increased about eight fold whereas CatD_sc_ expression was not altered (Fig. 3a). The elevated protein level of CatD_hc_ is in line with a significantly enhanced in vitro enzymatic activity (Fig. 3b). A second independent pool of MEF *Grn*^**−/−**^ as well as single cell clones additionally confirmed altered maturation and elevated levels of CatD_hc_ (Additional file 1: Figure S2c, d). Similar to mouse brain, CatB and CatL showed altered maturation (Fig. 3c, e) and a robust increase of in vitro activity (Fig. 3d, f). Low amounts of stably expressed PGRN were sufficient to rescue altered maturation of CatD and to lower hyperactivity of cathepsins (Fig. 3g, Additional file 1: Figure S3a, b). Thus, MEF, like total brain extract of *Grn*^**−/−**^ mice, exhibit increased cathepsin expression, maturation and in vitro activity. To investigate the functional consequences of enhanced lysosomal activity we compared general protein degradation in *Grn*^**−/−**^ and *Grn*^+/+^ MEF. Newly synthesized proteins were metabolically pulse labeled for one hour and chased for indicated periods of time (Fig. 4a). The relative protein turnover calculated by the percentage of remaining radiolabeled protein was higher in *Grn*^**−/−**^ MEF compared to *Grn*^+/+^ MEF (Fig. 4a). In particular, during the first 24 h protein degradation is elevated in *Grn*^**−/−**^ MEF (Fig. 4a). Elevated lysosomal protein turnover in PGRN deficient MEF was also indicated by lower steady state levels of proteins degraded by lysosomes such as APP and its CTF [54–57] (Fig. 4b), while the *App* mRNA level is not altered in *Grn*^**−/−**^ MEF (Fig. 4c). To address the question whether autophagy is altered by PGRN deficiency, we analyzed ubiquitin and the adapter protein p62/SQSTM1 levels in *Grn*^−/−^ MEF. Interestingly, we detect reduced levels of ubiquitinated proteins and p62 in Grn^−/−^ MEF compared to *Grn*^+/+^ MEF but no change in autophagy marker LC3I and LC3II (Fig. 4d). Thus, we do not find evidence that enhanced autophagosome formation contributes to the enhanced lysosomal degradation in *Grn*^−/−^ MEF (Fig. 4a). Having observed an increased activity of cathepsins as well as enhanced lysosomal protein degradation in *Grn*^**−/−**^ MEF, we asked whether PGRN or the proteolytically generated granulin peptides are direct inhibitors of lysosomal cathepsins. To do so, recombinant PGRN, granulin peptides generated by elastase digestion of PGRN or recombinant granulin E were added to in vitro cathepsin activity assays using lysates derived from *Grn*^**−/−**^ MEF (Additional file 1: Figure S4). None of the PGRN variants added to the in vitro assays had a significant effect on proteolytic activity suggesting that cathepsins may not be directly inhibited by an interaction with PGRN or the granulin peptides.

### Selective impairment of lysosomal processing of cathepsins in microglia upon PGRN deficiency

**Fig. 5:**
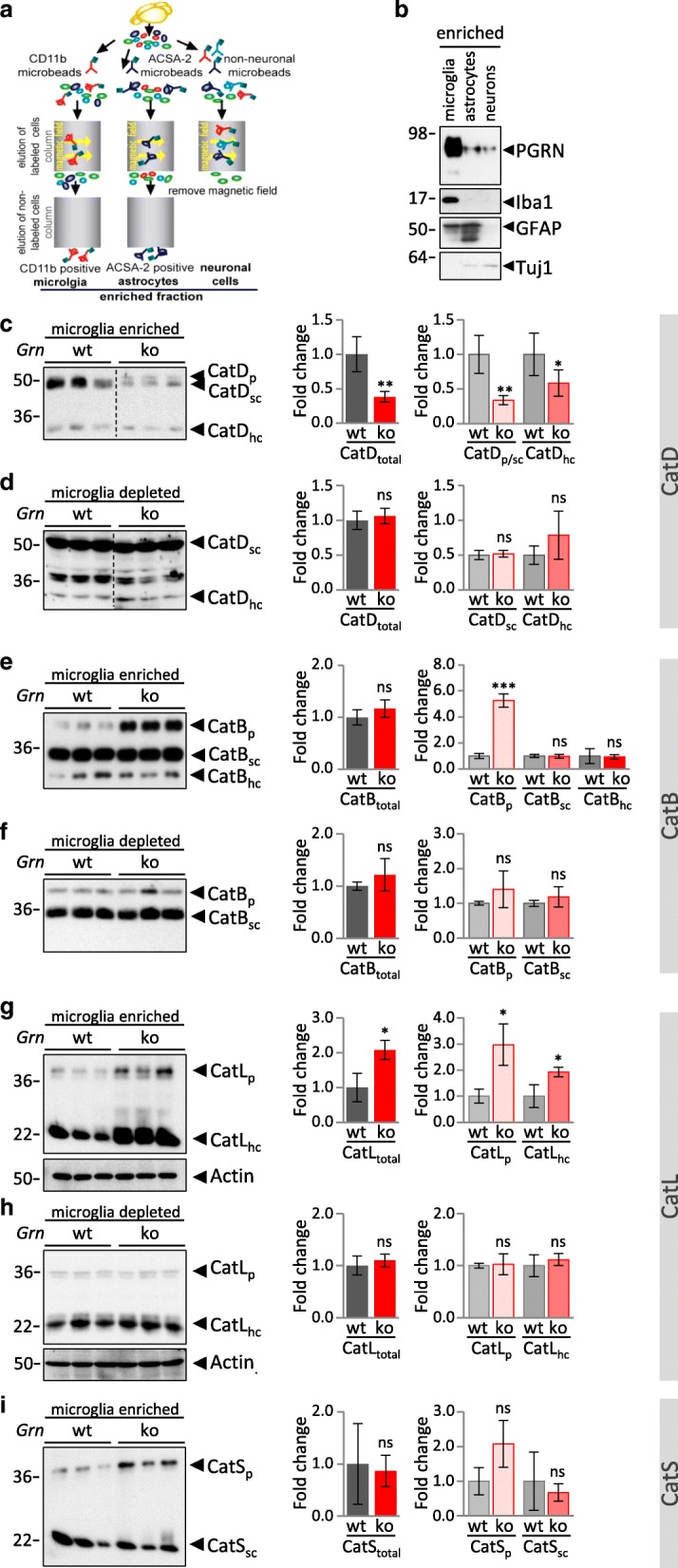
Cathepsin maturation is selectively impaired in *Grn*^−/−^ microglia. **a** Schematic representation of the brain cell isolation using MACS Technology (Miltenyi Biotec) **b** PGRN expression in acutely isolated microglia, astrocytes and neurons enriched fractions of 4- month-old wt mice detected by immunoblotting. The identity of neural cell types was verified by detection of Iba1 for microglia, GFAP for astrocytes and Tuj1 for neurons. **c-i** Cathepsin expression and maturation in the CD11b-positive, microglia enriched, fraction and the CD11b-negative, microglia depleted cellular fraction isolated form cortices of brain from 3-month-old *Grn*^+/+^ (wt) and *Grn*^**−/−**^ (ko) mice. Representative immunoblots for the cathepsin expression of CatD (**c, d**), CatB (**e, f**), CatL (**g, h**) and for CatS (**i**) (only microglia enriched fraction). The molecular weight standards in kilo Daltons (kDa) are indicated on the left side of all immunoblots. A dotted line in the blot indicates that samples of heterozygous mice were cut out, but all samples were loaded on one gel. Quantification of immunoblots for total cathepsin or maturation variants pro-form (p), single chain (sc) and heavy chain (hc) normalized to wt are shown as mean ± SD. For statistical analysis the unpaired, two-tailed student’s t-test was used to compare ko to wt mice (n = 3–5) (*, *p* < 0.05; **, *p* < 0.01; ***, *p* < 0.001; ns, not significant) (**c-i**)

**Fig. 6:**
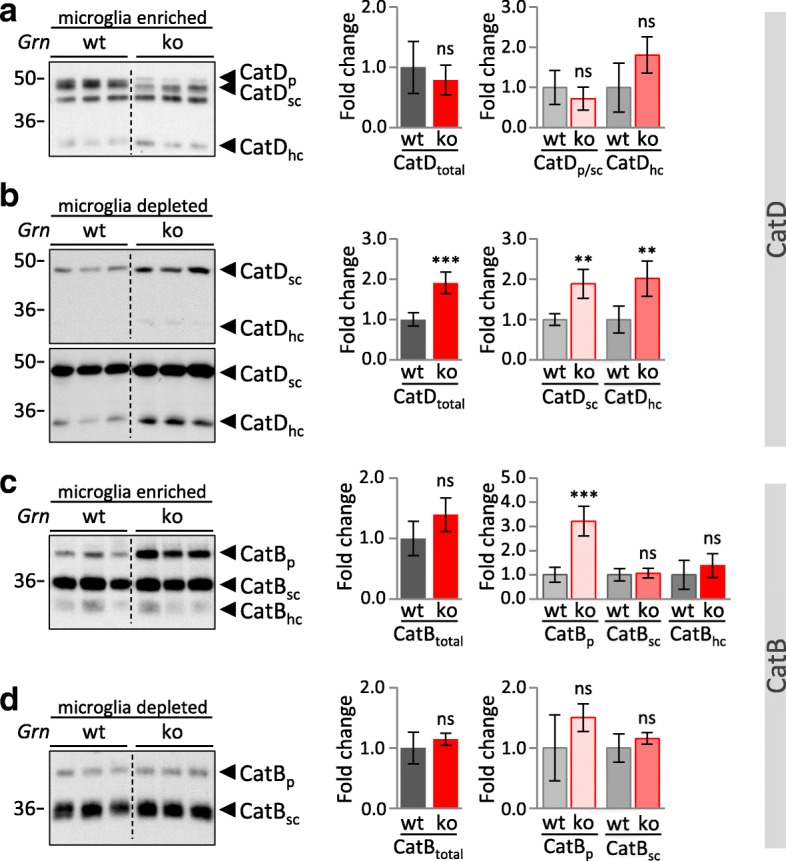
Cathepsins are elevated in the microglia depleted cell fraction of aged *Grn*^−/−^ mice. **a-d** Cathepsin expression and maturation in the CD11b-positive, microglia enriched, fraction and the CD11b-negative, microglia depleted cellular fraction isolated form cortices of brains from 12-month-old *Grn*^+/+^ (wt) and *Grn*^**−/−**^ (ko) mice. Representative immunoblots for the expression of CatD (**a, b**), CatB (**c, d**). The molecular weight standards in kilo Daltons (kDa) are indicated on the left side of all immunoblots. A dotted line in the blot indicates that samples of heterozygous mice were cut out, but all samples were loaded on one gel. Quantification of immunoblots for total cathepsin or pro-form (p), single chain (sc) and heavy chain (hc) normalized to wt are shown as mean ± SD. For statistical analysis the unpaired, two-tailed student’s t-test was used to compare ko to wt mice (*n* = 3–5) (*, *p* < 0.05; **, *p* < 0.01; ***, *p* < 0.001; ns, not significant) (**a-d**)

**Fig. 7:**
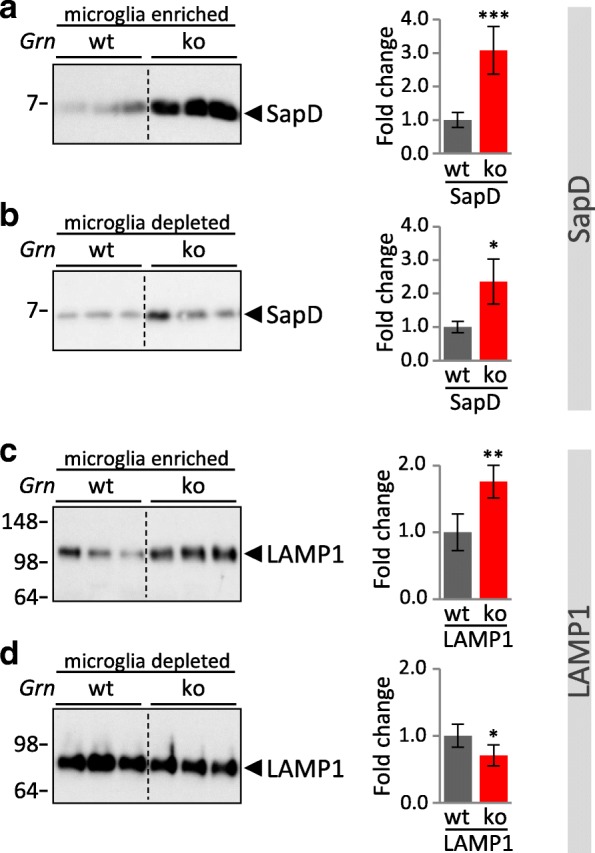
Enhanced accumulation of lysosomal proteins in microglia of 3-month-old *Grn*^−/−^ mice. Representative blots of LAMP1 (**a, b**) and saposin D (SapD) (**c, d**) in the CD11b-positive, microglia enriched fraction and the CD11b-negative, microglia depleted fraction isolated form brain cortices of 3-month-old *Grn*^+/+^ (wt) and *Grn*^**−/−**^ (ko) mice. A dotted line in the blot indicates that samples of heterozygous mice were cut out, but all samples were loaded on one gel. Data were normalized to the corresponding mean value of wt mice and are shown as mean ± SD. For statistical analysis the unpaired, two-tailed student’s t-test was used to compare ko to wt mice (*n* = 4–5) (*, *p* < 0.05; **, *p* < 0.01; ***, *p* < 0.001; ns, not significant)

Enhanced lysosomal activity of cathepsins in *Grn*^**−/−**^ MEF and total brain homogenates of *Grn*^**−/−**^ mice is contradictory to impaired protein degradation and accumulation of lipofuscin and NCL storage components caused by PGRN deficiency. Therefore, we analyzed the consequence of PGRN deficiency in microglia, which are known to express more than 50-fold higher levels of *Grn* mRNA as compared to neurons [58, 59]. We hypothesized that PGRN depletion may cause cell autonomous effects in microglia, which could be different to the majority of the non-microglial brain cell population. First, to confirm predominant PGRN protein expression in microglia, we performed immunoblots on lysates of acutely isolated microglia, astrocytes and neurons from adult wildtype mouse brain (Fig. 5a). This fully confirmed that PGRN is most robustly expressed in microglia (Fig. 5b). To address whether PGRN deficiency results in cell autonomous alterations of lysosomal function in microglia, we analyzed protein expression and maturation of selected cathepsins in acutely isolated microglia. Strikingly, microglia isolated from 3-month-old *Grn*^**−/−**^ mice show impaired maturation of cathepsins and an accumulation of inactive pro-forms of CatD, CatB, CatL and CatS (Fig. 5c, e, g, i). For CatB and CatL, the pro-form is significantly elevated (Fig. 5e, g). The relative increase of CatD_p_ in *Grn*^**−/−**^ could not be quantified since CatD_p_ cannot be detected in *Grn*^+/+^ microglia. Despite a general increase of pro-cathepsins, the levels of mature cathepsins are differentially affected by the loss of PGRN. Thus, the total protein level of CatD and the potentially active forms CatD_sc_ and CatD_hc_ are significantly reduced in microglia of *Grn*^**−/−**^ (Fig. 5c). However a significantly reduced in vitro activity of CatD could not be detected (Additional file 1: Figure S5a). For the other analyzed cathepsins the total level is unchanged (CatB, CatS) (Fig. 5e, i) or even elevated (CatL) (Fig. 5g). In the microglia depleted fraction isolated from 3-month-old mice, no significant differences of cathepsin maturation, expression level or activity between *Grn*^**−/−**^ and *Grn*^+/+^ can be observed (Fig. 5d, f, h; Additional file 1: Figure S5a), whereas in the microglia depleted fraction of 12-month-old mice CatD is significantly elevated in *Grn*^**−/−**^ (Fig. 6b) which results in enhanced CatD activity (Additional file 1: Figure S5b). However, in microglia of 12-month-old *Grn*^**−/−**^ mice altered expression levels of CatD and CatB are not further enhanced but rather slightly reduced (Fig. 6a, c), e.g. immature CatB_p_ shifts from an almost 6-fold increase in 3-month-old mice to an 3-fold increase in 12-month-old mice (Fig. 6c). In line with altered processed or reduced lysosomal cathepsins specifically in microglia, LAMP1 and saposin D accumulation occurred exclusively (LAMP1) or more robust (saposin D) in the microglia enriched fraction compared to the microglia depleted fraction isolated from 3-month-old mice (Fig. 7a-c).

Thus our data indicate that microglia and the remaining neural cell populations show fundamentally different lysosomal phenotypes upon PGRN deficiency.

## Discussion

Accumulating evidence suggests that impaired lysosomal protein degradation plays a major role in FTLD-TDP [15]. Lysosomal dysfunction seems to be specifically associated with FTLD-TDP caused by *GRN* haploinsufficiency [18]. Furthermore total loss of PGRN leads to NCL (CLN11) [3], a lysosomal storage disease with severe neurodegeneration. However, it is still unknown if and how PGRN affects lysosomal homeostasis. Based on the selective expression of GRN in microglia (Fig. 5b) [51, 58, 59], we now searched for cell autonomous and non-cell autonomous deficits upon loss of PGRN.

We provide strong evidence that loss of PGRN selectively impairs lysosomal function in microglia. Microglia isolated from 3-month-old *Grn*^**−/−**^ mice showed strongly reduced CatD levels compared to microglia isolated from *Grn*^**+/+**^ mice, which surprisingly did not result in a significantly reduced in vitro activity. However, impaired maturation might not be reflected by the in vitro activity assay since defective CatD maturation and catalytic activity could be hidden by optimal in vitro conditions. In addition, maturation of CatB, CatL and CatS was impaired. In contrast, in the microglia depleted fraction, isolated from the same 3-month-old *Grn*^**−/−**^ mice, no altered cathepsin expression or maturation was observed. Furthermore, during ageing saposin D and LAMP1 accumulated earlier and to a higher extent in the microglia enriched fraction than in the microglia depleted fraction. Our finding that impaired maturation of lysosomal enzymes in microglia already occurs in early adulthood before other pathological hallmarks suggests that lysosomal dysfunction may be a primary consequence upon loss of PGRN expression. Thus, our findings suggest a cell autonomous reduction of lysosomal function caused by PGRN deficiency in microglia, which as a consequence appears to culminate during ageing in a compensatory upregulation of lysosomal activity selectively in non-microglial cells. Indeed, in the microglia depleted fraction isolated from aged *Grn*^**−/−**^ mice CatD single and heavy chain are 2-fold and CatD in vitro activity is 2.5-fold elevated compared to *Grn*^**+/+**^ mice. Our findings are supported by the observation that cultured neurons from *Grn*^**−/−**^ mice show enhanced lysosomal proteolysis [51]. Moreover, in brain tissue of FTLD-TDP patients CatD is accumulating in neurons [21]. In line with enhanced cathepsin expression in non-microglial cells, mRNA, protein levels and in vitro activities of CatD, CatB, and CatL were increased in total brain of aged *Grn*^**−/−**^ mice. Furthermore, in line with recent findings [21, 38, 40, 51, 59, 60], a subset of lysosomal proteases and membrane proteins were upregulated in 6- and 12-month-old *Grn*^**−/−**^ mice. In addition to altered cathepsin levels, we demonstrate altered proteolytic processing and maturation of CatD, CatB, and CatL in the microglia enriched fraction, total brain lysates and MEF of *Grn*^**−/−**^ mice. While in microglia proteolytic inactive pro-forms accumulate, potentially active single chain or heavy chain variants accumulate in total brain and MEF in accordance with increased in vitro activity. For example, robustly enhanced levels of the CatD_hc_ variant are observed in *Grn*^**−/−**^ MEF which is in line with findings by Tanaka et al. [21]. Previous research revealed enhanced [51, 59] as well reduced lysosomal enzyme activities [38, 39] in various *Grn*^**−/−**^ cells types or tissue. Based on our findings, these discrepancies may be explained by different cell types analyzed, difficulties with the determination of specific activities of lysosomal enzymes due to their complex proteolytic processing and consequences for their proteolytic activity [53, 61]. Single chain variants as well as dimeric variants of heavy and light chain are catalytically active whereas separated heavy and light chains are inactive [53, 61]. Since we cannot determine the amount of active species, we cannot calculate the specific activity. Indeed, in total brain of *Grn*^**−/−**^ mice the increase of CatD protein is much stronger than the increase in enzyme activity which might indicate reduced specific activity as previously shown for CatD [38, 39]. However, it is unlikely that PGRN directly affects the specific activity of lysosomal proteases, because in our hands, adding PGRN, elastase digested PGRN, or granulin E to the in vitro activity assays of CatD, CatB, and CatL did not alter their activity. This might indicate that PGRN most likely modulates maturation and turnover of cathepsins.

In MEF, enhanced cathepsin activities are reversible by low expression levels of PGRN. Rescue of the lysosomal phenotype of *Grn*^**−/−**^ by a very minor amount of PGRN is in line with recent data showing that low levels of AAV-expressed neuronal PGRN are sufficient to rescue lysosomal phenotypes of *Grn* knockout mice [62]. Moreover, this also provides additional support for the lack of lysosomal abnormalities in heterozygous, neuronal or incomplete microglial *Grn* knockout mouse models [63, 64]. Finally, elevated catalytic activities of cathepsins result in enhanced protein turnover in *Grn*^**−/−**^ MEF, which indicates enhanced protein degradation in lysosomes. In line with enhanced lysosomal degradation, levels of lysosomal targeted proteins such as mature APP and its CTF are significantly reduced in *Grn*^**−/−**^ MEF while LC3I and LC3II levels are unchanged. Only under cellular stress impaired autophagy or altered autophagic flux has been reported for bone marrow derived macrophages (BMDM) [40].

The cell type dependent effects of PGRN deficiency in microglial and non-microglial cells could be caused by different lysosomal gene expression signatures in microglia/monocytes compared to other brain cells. Microglia not only express more and higher levels of lysosomal enzymes, they also express more and higher levels of lysosomal enzyme inhibitors. Thus regulation of lysosomal activity might be more complex in microglia. Further work needs to be done to elucidate how PGRN modulates cathepsin activities and which role lysosomal generated granulins play [31, 32]. With this work we provide strong evidence that PGRN plays a role as modulator of lysosomal activity by affecting maturation of lysosomal cathepsins. Such a function has been suggested for granulin-like domains located at the C-terminus of papain-like cysteine protease in plants [65–67]. Here, the granulin domain might slow maturation of the protease. Furthermore, the granulin domain must be proteolytically removed to allow full maturation [68, 69].

Finally, the divergent effects of PGRN deficiency in microglial and non-microglial cells not only provide evidence for differential cell autonomous and non-autonomous activities of PGRN, but also suggest a crosstalk of microglia with other cell types throughout the brain. Interestingly, we [70] and others [71] previously found that microglial loss-of-function mutations in *TREM2* affect energy metabolism throughout the entire brain. Again, a rather small percentage of brain cells seemed to influence metabolism throughout the entire brain.

## Conclusions

**Fig. 8:**
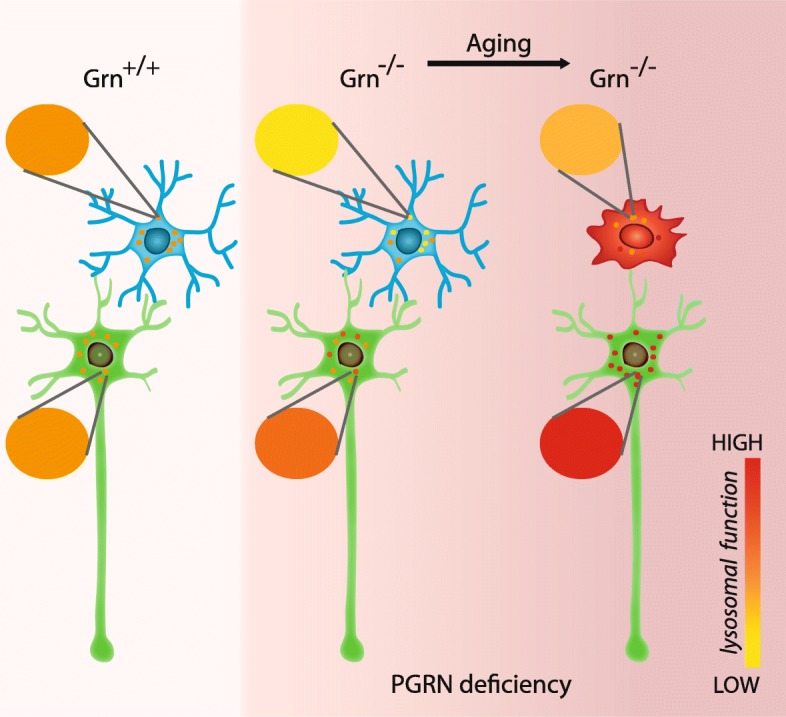
Schematic summary of differential effects of PGRN deficiency on brain cell types. Lysosomal impairment in microglia observed in young Grn^−/−^ mice result in enhanced lysosomal activity in non-microglial brain cells like neurons in aged Grn^−/−^ mice. Basal lysosomal functions of microglia and neurons are indicated in orange, changes to lower activity (yellow) in microglia or higher activity (red) in neurons or other non-microglial brain cells are caused by PGRN deficiency and aging

We conclude that PGRN deficiency leads to cell autonomous altered maturation and turnover of lysosomal cathepsins with cell type dependent differences and consequences. In particular in microglia, PGRN deficiency results in accumulation of inactive cathepsin pro-forms, while in other brain cells and in MEF, variants with increased catalytic activities were found. We speculate that impaired lysosomal function in microglia caused by PGRN deficiency already in young adults is likely responsible for impaired protein degradation and lipofuscin accumulation. With aging of *Grn*^**−/−**^ mice, non-microglial brain cells try to compensate decreased protein degradation by microglia, with enhanced expression of selective cathepsins as shown for CatD, CatB and CatL (Fig. 8).

## Additional files

Additional file 1: **Figure S1.** Elevated transcript levels of cathepsins in aged *Grn*^−/−^ mice. **Figure S2.** PGRN loss results in accumulation of LAMP1 and saposin D in MEF. **Figure S3.** Altered maturation of CatD and activity of cathepsins can be rescued by stable PGRN expression. **Figure S4.** PGRN, elastase digested PGRN and granulin E do not affect in vitro activity of cathepsins. **Figure S5.** Selectively enhanced CatD in vitro activity in non-microglial brain cells of aged *Grn*^−/−^ mice. (PPTX 1753 kb)

Additional file 2: Table S1. mRNA expression of selected genes associated with the lysosome-autophagy degradation pathway in brain of Grn+/+ and Grn−/− mice at 6 and 12 months of age. (XLSX 30 kb)

## Abbreviations

APP: Amyloid precursor protein
BMDM: Marrow derived macrophages
CatB: Cathepsin B protein
CatD: Cathepsin D protein
CatL: Cathepsin L protein
CatS: Cathepsin S protein
CNS: Central nervous system
CTF: C-terminal fragment
*Ctsb*: Mouse cathepsin B gene
*Ctsd*: Mouse cathepsin D gene
*Ctsl*: Mouse cathepsin L gene
FTLD: Frontotemporal lobar degeneration
*GRN*: Human progranulin gene
*Grn*: Mouse progranulin gene
hc: Heavy chain
*Hexb*: Hexosaminidase subunit b
MEF: Mouse embryonic fibroblasts
NCL: Neuronal ceroid lipofuscinosis
p: Pro-form
PGRN: Progranulin protein
pp: Pre-proform
qRT-PCR: Quantitative reverse transcription-polymerase chain reaction
sc: Single chain
SLPI: Secretory leucoprotease inhibitor
TDP-43: TAR DAN binding protein-43
TFEB: Transcription factor EB
