# Supplementary material for: Early lysosomal maturation deficits in microglia triggers enhanced lysosomal activity in other brain cells of progranulin knockout mice
Source: Mol Neurodegener. 2018 Sep 4;13:48. doi: 10.1186/s13024-018-0281-5 (PMC6123925; doi:10.1186/s13024-018-0281-5)
Supplement: Supplementary file 1 — Figure S1. Elevated transcript levels of cathepsins in aged Grn−/− mice. Figure S2. PGRN loss results in accumulation of LAMP1 and saposin D in MEF. Figure S3. Altered maturation of CatD and activity of cathepsins can be rescued by stable PGRN expression. Figure S4. PGRN, elastase digested PGRN and granulin E do not affect in vitro activity of cathepsins. Figure S5. Selectively enhanced CatD in vitro activity in non-microglial brain cells of aged Grn−/− mice. (PPTX 1753 kb) [file 13024_2018_281_MOESM1_ESM.pptx]

## Slide 1
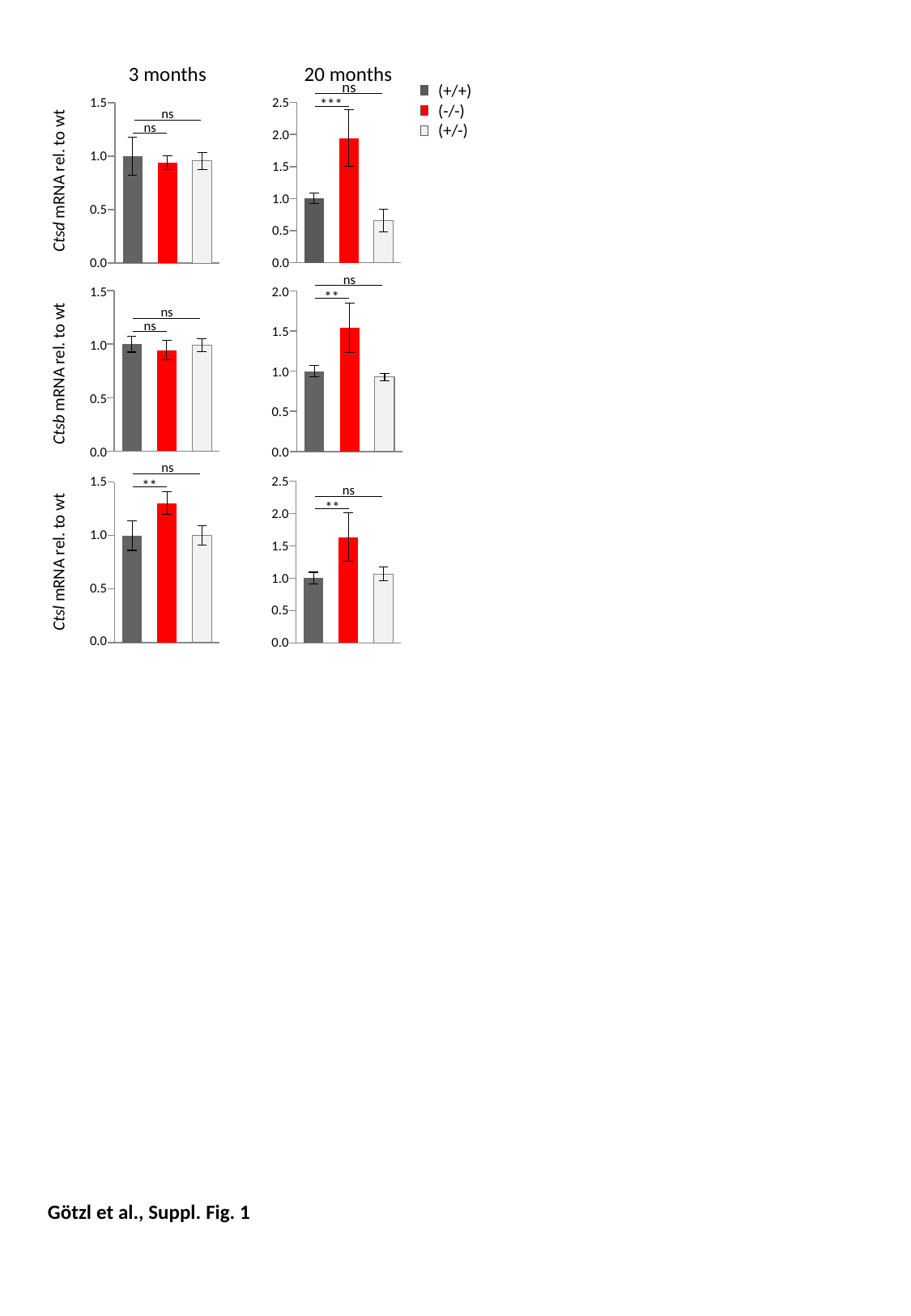

3 months
20 months
ns
***
(+/+)
(-/-)
(+/-)
### Chart
| Category | |
|---|---|
### Chart
| Category | |
|---|---|1.5
2.5
ns
ns
2.0
1.0
1.5
Ctsd mRNA rel. to wt
1.0
0.5
0.5
0.0
0.0
ns
**
### Chart
| Category | |
|---|---|
### Chart
| Category | |
|---|---|1.5
2.0
ns
ns
1.5
1.0
1.0
Ctsb mRNA rel. to wt
0.5
0.5
0.0
0.0
ns
**
### Chart
| Category | |
|---|---|
### Chart
| Category | |
|---|---|1.5
2.5
ns
**
2.0
1.0
1.5
Ctsl mRNA rel. to wt
1.0
0.5
0.5
0.0
0.0
Götzl et al., Suppl. Fig. 1

## Slide 2
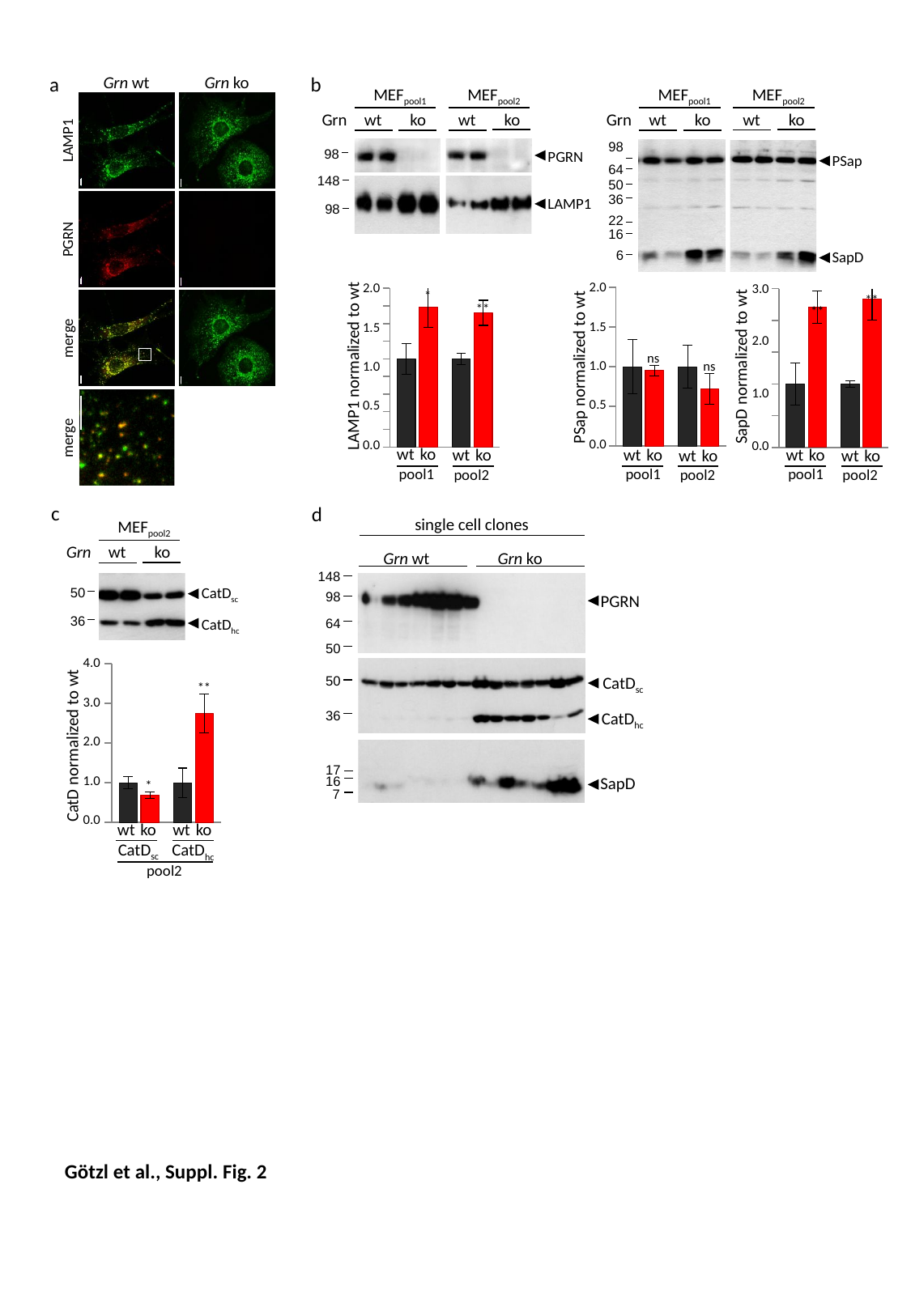

a
b
MEFpool2
 wt
ko
MEFpool1
Grn
 wt
ko
98
PSap
64
50
36
22
16
SapD
6
MEFpool2
 wt
ko
MEFpool1
Grn
 wt
ko
98
PGRN
148
LAMP1
98
Grn ko
Grn wt
LAMP1
PGRN
merge
merge
2.0
### Chart
| Category | | |
|---|---|---|1.5
LAMP1 normalized to wt
1.0
0.5
0.0
wt
ko
wt
ko
pool1
pool2
2.0
### Chart
| Category | | |
|---|---|---|1.5
PSap normalized to wt
1.0
0.5
0.0
wt
ko
wt
ko
pool1
pool2
3.0
### Chart
| Category | | |
|---|---|---|2.0
SapD normalized to wt
1.0
0.0
wt
ko
wt
ko
pool1
pool2
*
**
**
**
ns
ns
c
d
single cell clones
Grn wt
Grn ko
148
98
PGRN
64
50
CatDsc
50
36
CatDhc
17
SapD
16
7
MEFpool2
Grn
 wt
ko
CatDsc
50
36
CatDhc
4.0
3.0
2.0
CatD normalized to wt
1.0
0.0
### Chart
| Category | | |
|---|---|---|wt
ko
wt
ko
pool2
CatDsc
CatDhc
**
*
Götzl et al., Suppl. Fig. 2

## Slide 3
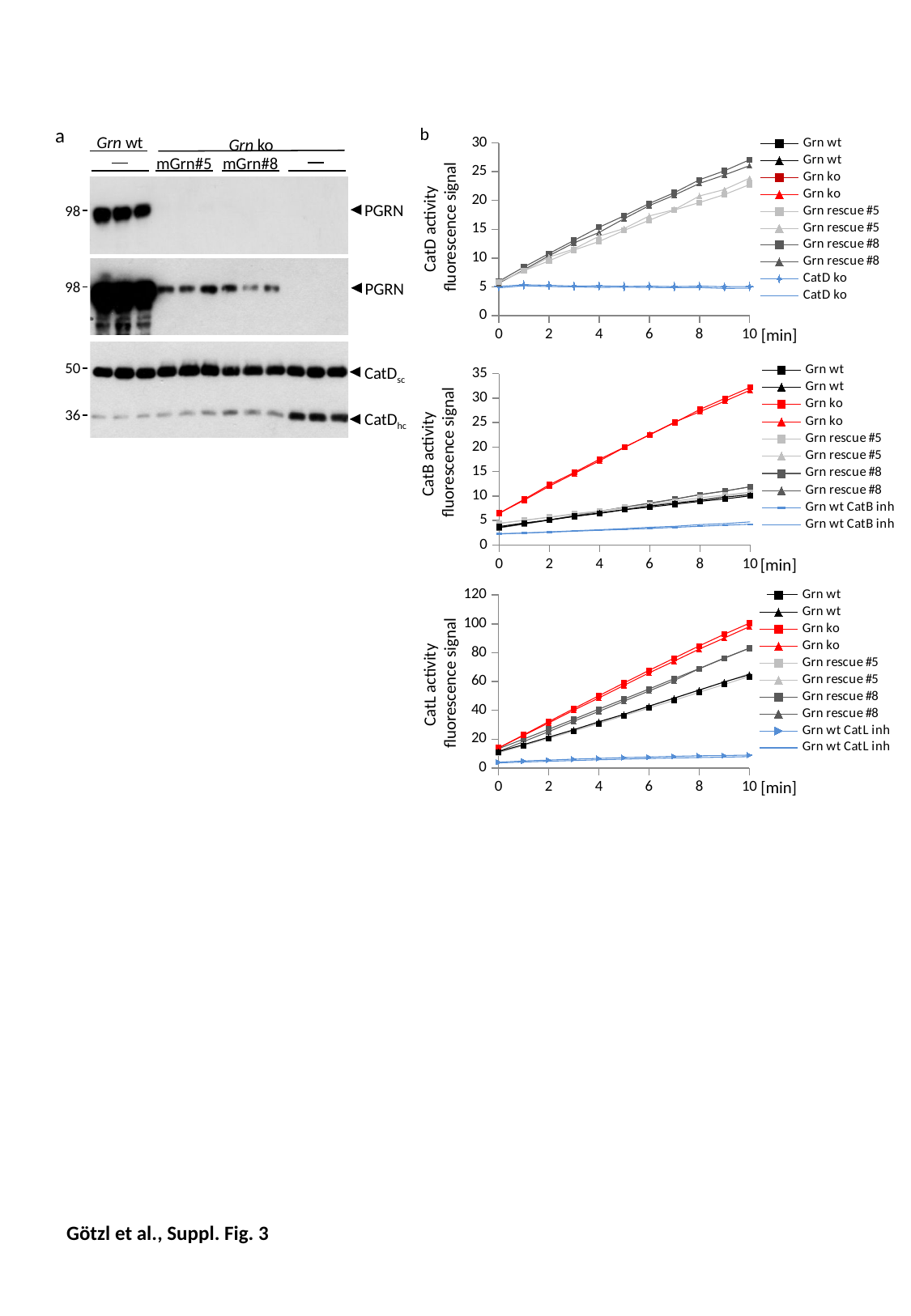

a
b
Grn ko
Grn wt
mGrn#5
mGrn#8
98
98
50
36
PGRN
PGRN
CatDsc
CatDhc
### Chart
| Category | | | | | | | | | | |
|---|---|---|---|---|---|---|---|---|---|---|CatD activity
 fluorescence signal
[min]
### Chart
| Category | | | | | | | | | | |
|---|---|---|---|---|---|---|---|---|---|---|CatB activity
 fluorescence signal
[min]
### Chart
| Category | | | | | | | | | | |
|---|---|---|---|---|---|---|---|---|---|---|CatL activity
 fluorescence signal
[min]
Götzl et al., Suppl. Fig. 3

## Slide 4
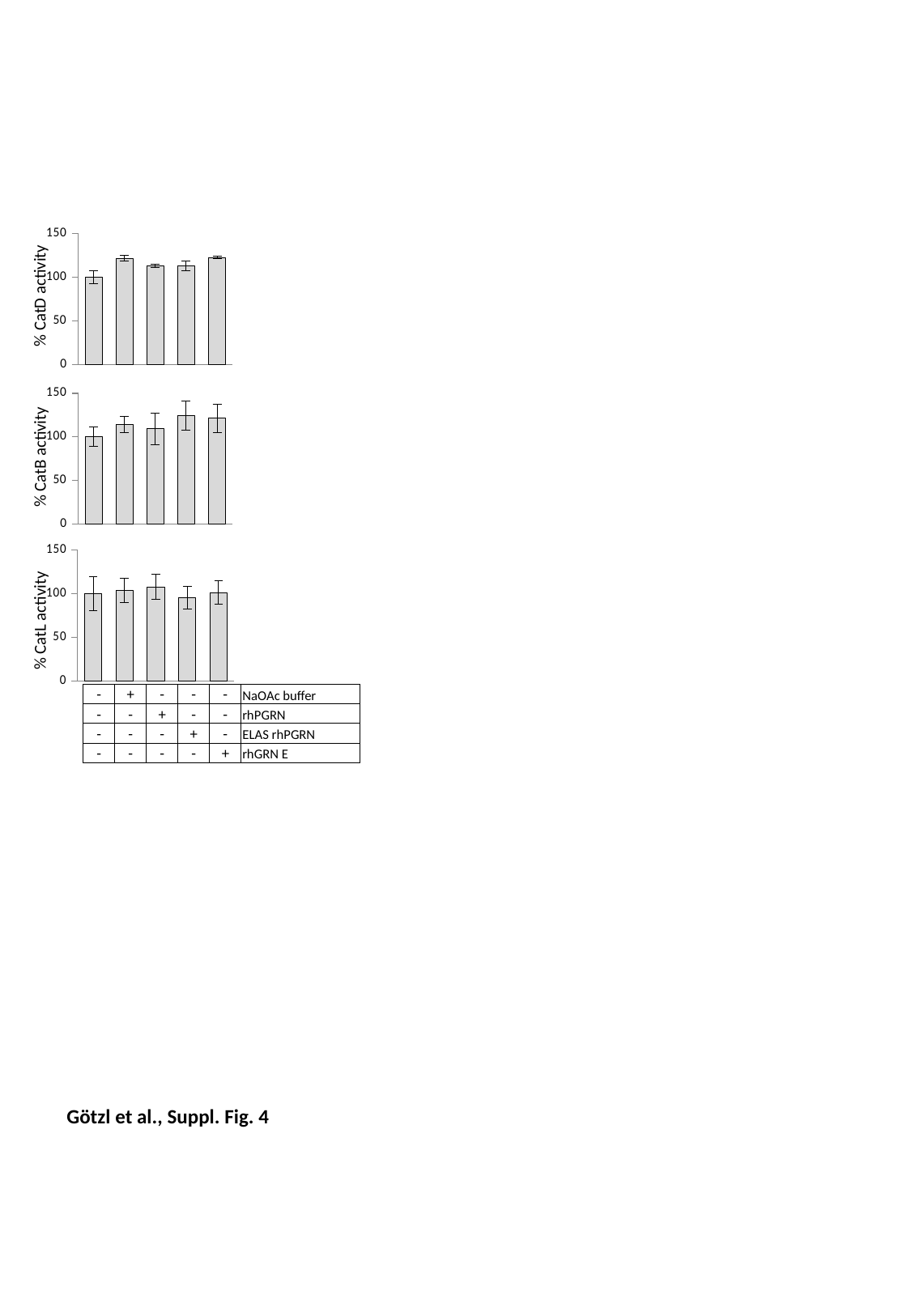

### Chart
| Category | |
|---|---| % CatD activity
### Chart
| Category | |
|---|---| % CatB activity
### Chart
| Category | |
|---|---|
| control | 100.0 |
| buffer | 103.29945842874055 |
| PGRN | 107.26929240697962 |
| PGRN elast | 95.11507383849093 |
| GRN E | 100.82836155709182 |% CatL activity
| - | + | - | - | - | NaOAc buffer |
| --- | --- | --- | --- | --- | --- |
| - | - | + | - | - | rhPGRN |
| - | - | - | + | - | ELAS rhPGRN |
| - | - | - | - | + | rhGRN E |
Götzl et al., Suppl. Fig. 4

## Slide 5
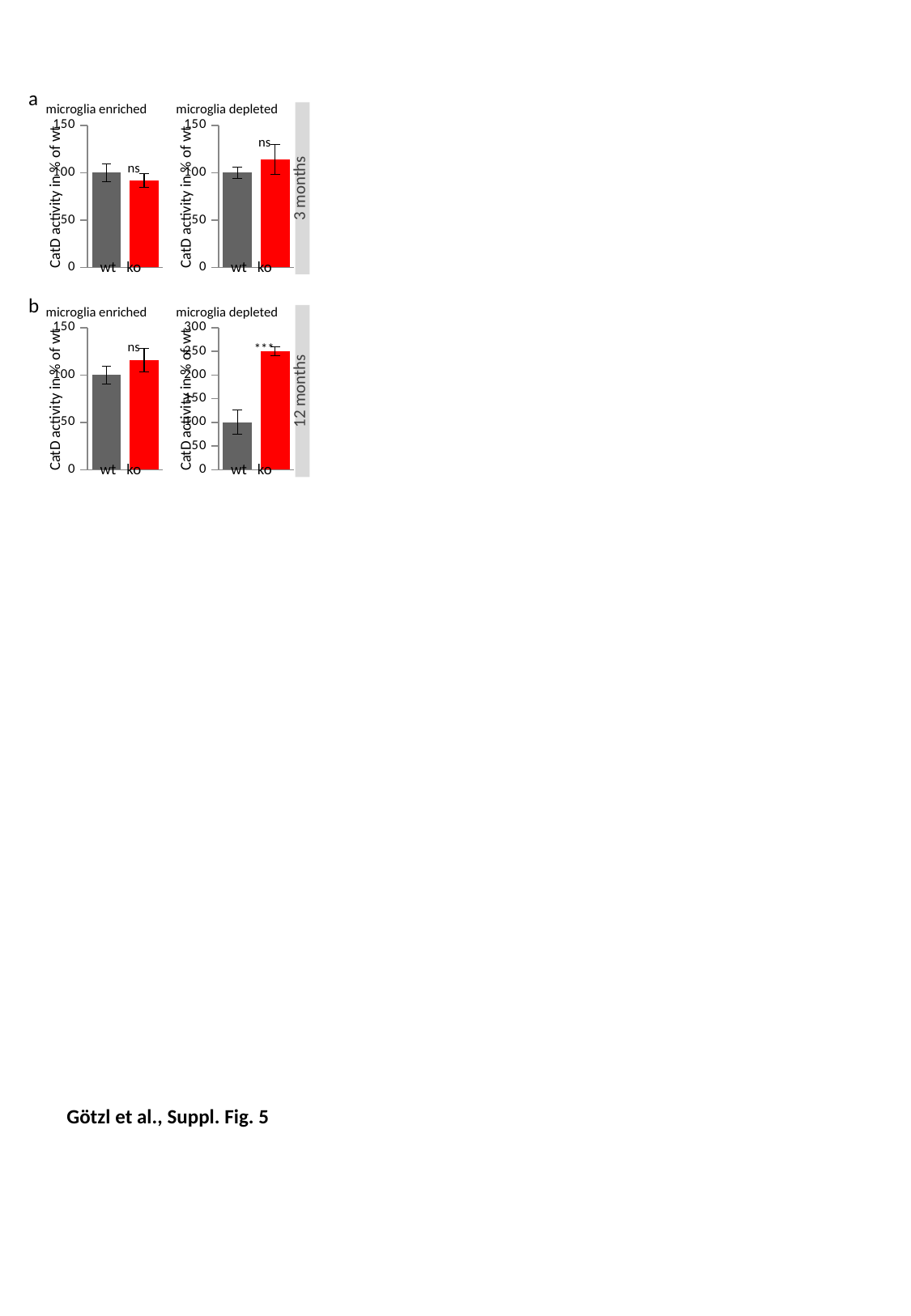

a
microglia enriched
microglia depleted
3 months
### Chart
| Category | |
|---|---|
### Chart
| Category | |
|---|---|ns
ns
 CatD activity in % of wt
 CatD activity in % of wt
wt
ko
wt
ko
b
microglia enriched
microglia depleted
12 months
### Chart
| Category | |
|---|---|
### Chart
| Category | |
|---|---|ns
***
 CatD activity in % of wt
 CatD activity in % of wt
wt
ko
wt
ko
Götzl et al., Suppl. Fig. 5
